# Supplementary material for: Achieving Quality and Effectiveness in Dementia Using Crisis Teams (AQUEDUCT): a randomised controlled trial evaluating the impact of a best practice Resource Kit used by teams managing crisis in dementia
Source: Nat Commun. 2025 Jul 11;16:6414. doi: 10.1038/s41467-025-61537-z (PMC12254196; doi:10.1038/s41467-025-61537-z)
Supplement: Supplementary file 1 — Supplementary Information [file 41467_2025_61537_MOESM1_ESM.pdf]

Supplementary Material

Table S1. Primary outcome complete case and sensitivity analyses results: Estimated Incidence Rate Ratios (IRR) using a negative binomial model, 95% confidence interval and p-values for the difference between intervention and control arm

	N	IRR	p-value	95% confidence interval	
a. Complete case primary outcome results					
Difference in psychiatric hospital admissions at six months (constituency-level population with dementia as offset)	22	0.75	0.37	1.54	0.434
b. ITT sensitivity analyses results					
Using baseline psychiatric hospital admissions as offset	23	1.18	0.78	1.18	0.435
Using constituency-level population with dementia as offset and removing the outlier (more than 150 psychiatric hospital admissions in six months)	23	0.78	0.31	1.99	0.598
Using constituency-level population with dementia as offset and using psychiatric hospital admissions at baseline as covariate	23	0.96	0.62	1.48	0.856
c. Complete-case sensitivity analyses results					
Using baseline psychiatric hospital admissions as offset	22	1.20	0.81	1.79	0.367
Using constituency-level population with dementia as offset and removing the outlier (more than 150 psychiatric hospital admissions in six months)	21	1.26	0.68	2.35	0.459
Using constituency-level population with dementia as offset and using psychiatric hospital admissions at baseline as covariate	22	0.98	0.64	1.52	0.935

Note: The intervention arm is the reference category in all trial results.

Figure S1. Summary of primary outcome results: Intention-to-treat, complete case, and sensitivity for the difference between arms in psychiatric hospital admissions at six months.

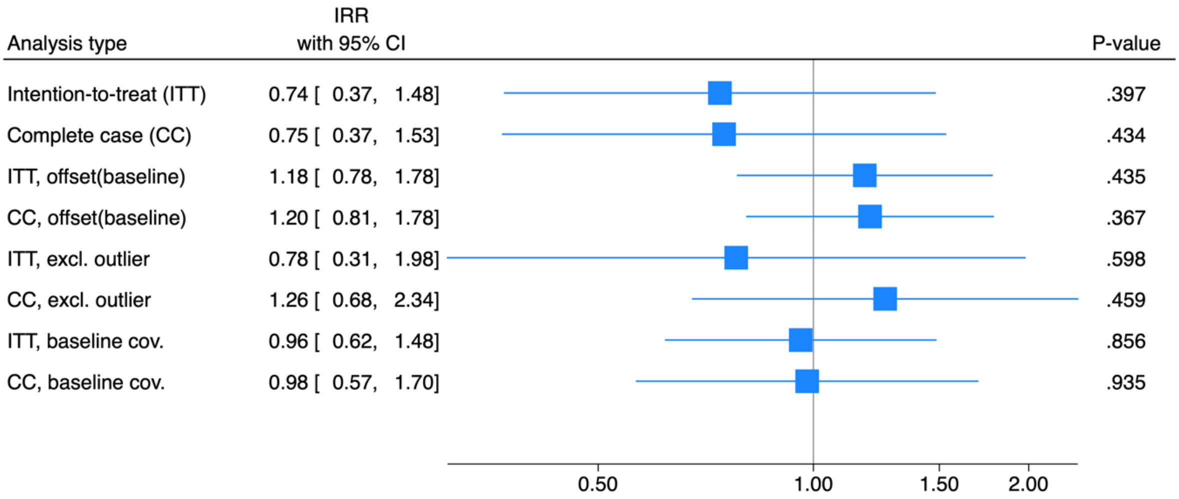

Table S2. Sensitivity analysis for secondary outcomes related to people with dementia and their carers (Coeff.), 95% confidence interval and p-values for the difference between intervention and control arm

	N	Coeff.	p-value	95% confidence interval	
a. Setting as missing scores and scales with more than 50% missing items					
General Health Questionnaire score (GHQ-12)	68	1.92	0.227	-1.22	5.07
Client Satisfaction Questionnaire score (CSQ-8)	73	-2.00	0.189	-5.01	1.01
b. Adjusted by gender					
General Health Questionnaire score (GHQ-12)	56	1.48	0.447	-2.40	5.37
Client Satisfaction Questionnaire score (CSQ-8)	57	-1.00	0.539	-4.24	2.24a

Note: The intervention arm is the reference category in all trial results. When less than 50% of the items in the scale had missing values, they were imputed using pro-rating. The scale was set to missing for over 50% of the items with missing values. Quantile regression for CSQ-8 at the median (P50 = 29) with robust standard errors.

Table S3. Complete case secondary outcome analysis results for TMCD staff (Coeff.), 95% confidence interval and p-values for the difference between intervention and control arm

	N	Coeff.	p-value	95% confidence interval	
c. Complete case results					
General Health Questionnaire score (GHQ-12)	211	0.71	0.376	-0.86	2.29
Work Acceptance & Action Questionnaire score (WAAQ)	212	0.17	0.885	-2.14	2.48
Utrecht Work Engagement Scale (UWES)	215	-2.29	0.338	-6.98	2.40
d. Multiple imputation results adjusted by gender					
General Health Questionnaire score (GHQ-12)	204	1.15	0.252	-0.82	3.12
Work Acceptance & Action Questionnaire score (WAAQ)	204	-0.08	0.946	-2.50	2.34
Utrecht Work Engagement Scale (UWES)	204	-2.99	0.282	-8.42	2.45

Note: The intervention arm is the reference category in all trial results. In the complete case analysis, missing items in scores and scales were imputed using pro-rating. When 50% or more of the items had missing values, the final score was set to missing and imputed through Multilevel Multiple Imputation using the jomo R package and calculating p-values. There were 34 observations with missing values for gender, which were omitted from the adjusted by gender multiple imputation analysis.
